# Development of a Technology-Assisted Food Frequency Questionnaire for Elementary and Middle School Children: Findings from a Pilot Study

**DOI:** 10.3390/nu11051103

**Published:** 2019-05-17

**Authors:** Andrea L. Deierlein, Jessica D. Bihuniak, Ekanta Nagi, Jackie Litvak, Christian Victoria, Tanya Braune, Rick Weiss, Niyati Parekh

**Affiliations:** 1 Division of Public Health Nutrition, School of Global Public Health, New York University, New York, NY 10003, USA; jl7589@nyu.edu (J.L.); christian.victoria@nyu.edu (C.V.); tb1902@nyu.edu (T.B.); np30@nyu.edu (N.P.); 2 Department of Nutrition and Food Studies, Steinhardt School, New York University, New York, NY 10003, USA; jdb13@nyu.edu; 3 Department of Applied Statistics, Social Science, and Humanities, Steinhardt School, New York University, New York, NY 10003, USA; ekn235@nyu.edu; 4 Viocare Nutrition Software Solutions for Healthcare and Research, Princeton, NJ 08542, USA; weiss@viocare.com

**Keywords:** dietary assessment, food frequency questionnaire, children, adolescents, web-based

## Abstract

Background: This pilot study collected preliminary data for the modification of the VioScreen Food Frequency Questionnaire (FFQ), an adult-validated, self-administered, web-based dietary assessment tool for use in older children. Methods: A convenience sample of 55 children, aged 6–14 years, completed the VioScreen FFQ and 3-day diet record (reference standard). Caregivers completed a short sociodemographic questionnaire. Reported dietary intakes from the VioScreen FFQ and 3-day diet record were calculated using standard nutrient databases, and descriptive statistics were used to examine differences in food/beverage items and portion sizes between the two methods. Informal focus groups obtained user feedback and identified components of the VioScreen FFQ that required modifications. Results: The highest de-attenuated Pearson correlation coefficients between the VioScreen FFQ and 3-day diet record were observed for iron (r = 0.69), saturated fat (r = 0.59), and vegetables (r = 0.56), and the lowest were for whole grains (r = 0.11) and vitamin C (r = 0.16). Qualitative feedback was overall positive, and six technological modifications were identified. Conclusion: Findings from this pilot study provided valuable information on the process of evaluating the use of the VioScreen FFQ among older children, and will inform the future development of a modified version for this population.

## 1. Introduction

Diet plays an integral role in the physical growth, cognitive function, pubertal development, and overall health of children [1,2,3]. The consequences of poor diet during early life are long-lasting, with childhood nutritional status being a strong predictor of chronic diseases, including obesity, type 2 diabetes and cardio-metabolic disturbances in adolescence through adulthood [1]. Although there are several methods available to assess dietary intakes in adults, current dietary assessment tools available for self-administration in children are limited and rely mostly on parent/caregiver reports for children under 14 years [4,5]. Parental reports of children’s diets may be representative of intakes among toddlers and young children, but these reports are less accurate for elementary and middle school aged children [5].

Considering the importance of accounting for diet within pediatric clinical and epidemiological research, investigators require dietary assessment tools specifically designed for children which are valid, reliable, time-efficient, and reduce caregiver involvement. The food frequency questionnaire (FFQ) is a commonly used dietary assessment method that queries previous food and beverage intakes (usually during the previous month to one year) to estimate usual intakes. Several studies evaluated the validity of FFQs for use in adolescents [6]. To our knowledge, there is only one validated, self-administered FFQ assessing intakes within the previous month or longer for use in children and adolescents (ages 9–18 years) in the United States (U.S.)—the Youth and Adolescent Questionnaire (YAQ) [7]. The YAQ was developed by modifying an adult validated FFQ and has been used in several studies; however, it is over 20 years old and only available in a paper-based format [7]. The development of a technology-based, self-administered, and validated dietary assessment tool for children could improve large-scale dietary reporting accuracy and completion rates with less burden, and can be used across research, clinical, and community settings. Here, we present the findings from a pilot study to modify the VioScreen FFQ—a web-based, adult-validated FFQ—for use in older children (6–14 years) [8]. The objectives of this pilot study were to collect and compare preliminary data on children’s dietary intakes using the VioScreen FFQ and 3-day dietary records and to conduct informal focus groups with children regarding their experiences administering the VioScreen FFQ.

## 2. Materials and Methods 

### 2.1. Study Design

A convenience sample of children and adolescents were recruited from the New York University community and surrounding neighborhood area (New York, NY, USA) from September 2017–April 2018, using flyers, advertisements, social media posts, and word of mouth. Initial telephone screening interviews were conducted with caregivers of 61 children to determine eligibility: 6–14 years of age, fluent in English, did not consume primarily ethnic cuisines, and did not have dietary restrictions. Fifty-eight children met these criteria, and children and their caregivers were invited to attend an in-person study visit. During the visit, caregivers completed a short sociodemographic questionnaire. Research staff measured children’s heights to the nearest 0.1 cm (using a Seca wall stadiometer) and children’s weights to the nearest 0.1 kg (using a Seca digital scale). Children also self-administered the VioScreen FFQ. In the 7 days following the study visit, children completed a 3-day paper-based dietary record, which was used as a reference method [9]. Within approximately 1–2 months of the study visit, children were invited to participate in informal focus groups, which collected information regarding their experiences using the VioScreen FFQ. Children received a $25 gift card for completing both the VioScreen FFQ and 3-day dietary record and a $25 gift card for participating in the informal focus groups. Additionally, children received $5 per referral they provided who completed the study. This study was approved by the institutional review board at New York University. All parents/legal guardians and children provided informed consent/assent for completing the study visit and focus groups. 

### 2.2. VioScreen Food Frequency Questionnaire

**Figure 1:**
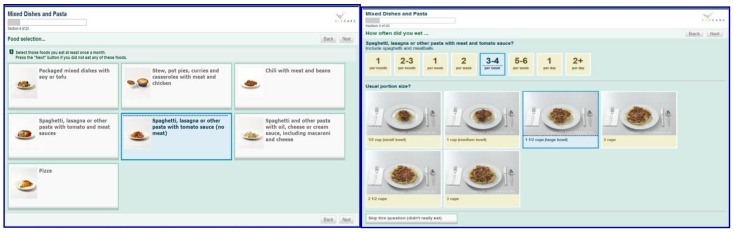
Examples of screens from the VioScreen food frequency questionnaire (FFQ) showing food selection choices and portion size options.

The VioScreen FFQ is a web-based dietary analysis software that was developed with grant funding from the National Institutes of Health, and has been used in both research and clinical settings [10]. The VioScreen FFQ uses graphics (approximately 1200 food images), branching questions (i.e., reduces missing foods and respondent burden), and up-to-date nutrition databases to generate detailed reports on nutrient intakes and food use patterns for the previous 90 days. It queries about 155 food and beverage items under 20 section headings: Cereals & Breads; Eggs & Meats; Chicken & Fish; Mixed Dishes & Pasta; Asian, Mexican & Soy Foods; Soups; Cheese & Dairy Products; Salads & Salad Vegetables; Garden Vegetables; Potatoes, Beans & Rice; Oil/Fat Used in Cooking; Sauces & Seasonings; Fruits; Sweets; Chips, Crackers & Snacks; Meal Replacement Drinks, Sports & Granola Bars; Milk, Coffee & Tea; Soft Drinks, Water & Juice; Alcoholic Beverages; and Supplements. Up to six graphical portion size options (i.e., different amounts of each food item displayed on a plate) are provided. The VioScreen FFQ has been scientifically validated for use in adults, with an approximate completion time of 20–25 min [8]. Example screen shots from the VioScreen FFQ of food item options and portion sizes are shown in Figure 1.

During the study visit, research staff provided children with a brief introduction to the VioScreen FFQ and a tutorial (a 5-min video produced by study staff) of how to use it. Children then self-administered the VioScreen FFQ using tablets under the supervision of research staff with minimal assistance (e.g., the section on Alcoholic Beverages was skipped). Caregivers were permitted to remain in the room and encouraged to allow children to independently complete the VioScreen FFQ. 

### 2.3. Three-Day Dietary Record

Upon completion of the study visit, children were provided with a 3-day paper-based dietary record to be returned to study staff within the following 7 days of the in-person visit. Children were given detailed instructions with a portion size estimation handout to record all foods and beverages consumed on two non-consecutive weekdays and one weekend day. Records requested information on food and beverage items, portion sizes, and time and location of meals. Research staff reviewed all dietary records and contacted children to clarify missing or incomplete information. The nutrient composition of all items recorded on the dietary records were calculated using the U.S. Department of Agriculture nutrient database [11]. 

### 2.4. Informal Focus Groups

Within two months following the in-person study visit, children who completed both the VioScreen FFQ and the 3-day dietary record were invited to participate in an informal focus group. There were two focus groups scheduled throughout the study period (January and April). Each focus group was scheduled for a weekend day, and was two hours in duration. During the focus group, research staff served as facilitators and reviewed the VioScreen FFQ with the children, asking them to identify questions or graphics that were confusing or they did not recognize, portion sizes that were too small or large, and missing food items. Focus groups were audio-recorded, transcribed, and evaluated by research staff to identify positive feedback and suggested improvements.

### 2.5. Statistical Analysis

Statistical analyses were conducted with RStudio (1.1.456) and with Stata SE version 15 (StataCorp LLC, College Station, TX, USA). Means, standard deviations, medians, and 10th–90th percentile ranges for energy, nutrient, and food group intakes were calculated for the VioScreen FFQ and the 3-day dietary records (intakes from 3-day dietary records were averaged across the three days). Differences in intakes between the two methods were compared with the Wilcoxon signed rank test. De-attenuated Pearson correlation coefficients were calculated between energy, nutrients, and food group values from the VioScreen FFQ and the mean of the 3-day dietary record [9]. De-attenuation accounts for the within-person variation from the multiple records. The formula used to calculate the de-attenuated Pearson correlation *(r_d_) was: r_d_ = r_o_ (V^2^_w_/V^2^_B_]/n)^0.5^*, where *r_o_* is the observed Pearson correlation, *n* is the number of days in the dietary record (*n* = 3), and (*V^2^_w_/V^2^_B_*) is the ratio of the within- to between-subject variances from repeated measures of nutrient/food group intakes. 

## 3. Results

### 3.1. Comparisons between Dietary Assessments

Fifty-five children (34 girls, 62%) participated in the study. All children completed the VioScreen FFQ and the 3-day dietary records. Characteristics of the children are shown in Table 1. Approximately half of the children were 6–10 years old (55%) and the majority were normal weight (80%). Most of the children were white (65%) and the remaining children identified as Asian (16%), Hispanic (11%), or Black (7%). The average completion time of the VioScreen FFQ was 29 min (standard deviation: 12 min). 

The highest de-attenuated Pearson correlations (≥0.50) between the VioScreen FFQ and the 3-day dietary records (i.e., reference standard) were observed for iron (0.69), saturated fat (0.59), vegetables (0.56), calories (0.52), carbohydrates (0.51), and sugar (0.50). The lowest correlations were observed for whole grains (0.11) and vitamin C (0.16), as seen in Table 2.

### 3.2. Informal Focus Groups 

There were 24 children (15 girls; mean age = 10.7 years, range 7–14) who participated in two informal focus group sessions (9 children in the first session and 15 children in the second session). Overall, the following topics emerged from these sessions: 

The VioScreen FFQ was well received and understood by the children. The written instructions were clear, the instructional video was helpful, and the visuals within the questionnaire were good. Some children mentioned that it had made them more mindful of what they were eating as well as of portion sizes. 

Some children felt that the questionnaire was too long and that they got bored/tired after some time. They suggested creating skip pathways to jump to specific sections. The questionnaire includes a place to skip to sections; however, it was not demonstrated in the instructions.

Children suggested providing examples in each group of food items and expanding to sub-groupings. For example, the “Tofu or soy breakfast sausage or other breakfast meats” category was confusing to the vegetarian children, as they were unsure if this was referring to breakfast meat alternatives or not. Also, sweet and savory breads were categorized together, and they felt that these should be separated. 

Missing foods pointed out by children included granola in the cereals section pomegranate in the fruits section, pickles, and not enough vegetarian options. 

The children were unclear on how to complete the questionnaire for non-home-prepared foods. For example, for smoothies, they were not thinking about these as separate components when completing the fruits section.

The children suggested the provision of a comments section for any additional foods or notes.

## 4. Discussion

The objective of this pilot study was to evaluate the use of the web-based adult-validated VioScreen FFQ to estimate dietary intakes among elementary- and middle-school-aged children. Correlation coefficients between children’s intakes reported on the VioScreen FFQ and the 3-day dietary records were moderate to low. The highest correlation coefficients, ranging from 0.50 to 0.68, were observed for calories, carbohydrates, saturated fat, iron, and vegetables, while the lowest correlations were observed for whole grains and vitamin C intakes. Informal focus groups revealed that children found the VioScreen FFQ easy to administer and enjoyed their overall experience with it. Several issues with the VioScreen FFQ were identified, including those with graphics, food groupings, portion sizes, and omitted food items, which would need to be modified prior to validating its use in children. 

Previous studies have evaluated the use of self-administered dietary assessment methods, including 24-h recalls, food records, and FFQs, to estimate intakes in older children and adolescents [2,6,12,13]. Among those studies that examined self-administration of FFQs, the majority did not include children younger than 12 years and used paper-based FFQs designed for populations outside of the U.S. [6]. Results from a meta-analysis of 16 studies that assessed the validation of paper-based FFQs (time intervals for intakes ranged from the previous week up to 1 year) among individuals 13–17 years old showed that pooled de-attenuated correlation coefficients for most nutrients/food groups ranged from 0.26 to 0.50, with the exception of calcium (0.55) and magnesium (0.51). The authors concluded that although most FFQs did not achieve absolute validity, they were adequately robust for ranking energy and nutrient intakes in adolescents. Studies that have evaluated web-based FFQs are limited and have only been conducted in non-U.S. countries [14,15]. Matthys et al. assessed the validity and reproducibility of a 69-item web-based FFQ (intakes during previous month) to estimate food group intakes among 104 Belgian adolescents (ages 12–18 years), with the 3-day estimated dietary records used as reference [14]. Spearman correlation coefficients were highest (>0.50) for breakfast cereals and soft drinks and lowest (<0.25) for pasta, potatoes, and vegetables. Similarly, Vereecken et al. investigated a 147-item web-based FFQ (intakes during previous month) among a convenience sample of 48 Belgian-Flemish adolescents (mean (SD) age, 14.6 (1.1) years), using a reference of four 24-h dietary recalls [15]. High correlation coefficients (>0.50) were observed for many nutrients and food groups, including energy, calcium, iron, and fat, while the lowest correlation coefficients (<0.25) were observed for fiber, fruits, and meats. Collectively, results from these studies and the current report suggest that web-based FFQs have the potential to collect valuable nutritional information from older children and adolescents. 

Correlation coefficients in the current pilot study were generally lower than those observed in previous studies, which may be at least partially attributable to differences in methodology, such as the reference method used (e.g., weighed dietary records, interviewer-administered 24-h recalls) and the FFQ. In the current study, the 3-day dietary records were not weighed, and were completed by the children. These records requested real-time detailed information on the types of food/beverage (e.g., brand, recipe ingredients), meal times, and locations, which children may not have accurately detailed. Additionally, the VioScreen FFQ queried about the intake of 155 items during the previous 3 months using graphics, portion sizes, and vocabulary tailored to U.S. adults, not younger populations. The informal focus groups conducted in the current study provided essential user feedback data from the target population, which will be used to direct future methodological improvements. Additional development studies and further pilot-testing are needed before the VioScreen FFQ can be used to assess the dietary intakes of U.S. children in research, clinical, and community settings. 

This pilot study provides valuable information regarding the process of evaluating the use of a self-administered web-based VioScreen FFQ among older children and determining necessary updates for its use in this population. We aimed to recruit children using informational flyers and pamphlets that were posted at various sites in neighborhoods throughout Manhattan, including libraries, museums, and kids’ clubs. However, the recruitment of children using this passive strategy was slow. Additional strategies were employed, including informational posts on parent/caregiver-specific social media groups and email Listservs, as well as actively recruiting through faculty at the New York University. Referral incentives (participants received an additional $5 gift card for each additional child they referred to the study) were also used to encourage participation. These strategies resulted in a diverse sample of children, all of whom completed the study.

The findings from the informal focus groups demonstrated that the VioScreen FFQ was well-received by the children and that they found this format easy to self-administer. As expected, children identified edits needed regarding the graphics used to depict certain food groups and certain omitted food items. For multiple sections of the VioScreen FFQ, children stated that they found the grouping of food items (and their corresponding graphics) to be confusing. For example, “peaches, plums and nectarines” were grouped together and the graphic only included peaches. In some instances, children reported being deterred from reporting their consumption of the food group (e.g., peaches, plums, or nectarines) if it included options that that they did not consume (e.g., they consume plums but they do not consume peaches); therefore, children suggested that food items should be separated into individual sub-groupings. Interestingly, some children reported that the FFQ made them feel more mindful of what they were eating as well as of portion sizes. It is not clear if or how this may have influenced the reporting of their dietary intake intakes. 

Limitations of the current pilot study, including the convenience sample of children living in New York City and the dietary reference method, may be addressed in a formal study with a larger sample size of at least 100–200 older children and adolescents [9]. Children’s characteristics, including overweight/obesity (greater child weight is associated with under-reporting), gender (boys may underestimate intakes), and age (older age improves accuracy) may influence the accuracy of self-reported dietary intakes and should be further investigated [16,17,18]. Additionally, during the focus groups, it was observed that older children (11–14 years) tended to actively participate more than younger children (ages 6–10 years), which suggests that groups should be separated to encourage participation among younger children. 

## 5. Conclusions

To our knowledge, there are no validated web-based FFQs to estimate nutrient and food group intakes in U.S. children and adolescents. This pilot study provides valuable information on the strategies and processes used to evaluate the use of an adult-validated web-based FFQ among a convenience sample of children aged 6–14 years old in New York City. The study revealed important modifications to tailor the questionnaire to children. Our immediate next step is to re-test and validate the modified FFQ for use in the pediatric population. 

## Figures and Tables

**Table 1:** Baseline characteristics of participants in the pilot study (*n* = 55).

Characteristic	*n*	%
Gender		
Boys	21	38
Girls	34	62
Age Category		
6 to 10	30	55
11 to 14	25	45
Race/Ethnicity		
White	36	65
Black	4	7
Hispanic	6	11
Asian	9	16
BMI Status		
Underweight (<5th)	2	4
Normal Weight (5th–<85th)	44	80
Overweight (85th–<95th)	5	9
Obese (≥95th)	4	7
Maternal Education Level		
Some College or Less	5	9
College Graduate	14	25
Greater than College	36	65
Number of away from home meals purchased (per week)		
<2	20	36
2–<4	26	47
≥4	9	17
Number of “family meals” (per week)		
0–<5	6	11
5–<10	22	40
10–<15	14	25
≥15	13	24

BMI: body mass index.

**Table 2:** Descriptive comparisons of nutrient and food group intakes estimated by the VioScreen food frequency questionnaire (FFQ) and 3-day dietary records among participants of the pilot study (*n* = 55).

	FFQ				3-day Food Record				*p* *	De-Attenuated Pearson Correlation Coefficients
	Mean	SD	Median	10th–90th Percentile	Mean	SD	Median	10th–90th Percentile		
Calories, kcal	1416.2	1111.3	1138.8	615.5, 2536.9	1740.9	572.8	1685.5	1110.8, 2502.0	<0.01	0.52
Carbohydrates, g	178.4	126.7	149.6	70.5, 318.3	225.9	82.1	220.8	137.5, 295.6	<0.01	0.51
Protein, g	53.5	41.4	43.0	21.3, 90.7	69.0	21.8	65.6	45.6, 103.1	<0.001	0.33
Fats, g	58.2	56.2	42.2	16.1, 102.9	65.2	24.3	59.6	41.4, 108.1	0.03	0.48
Saturated Fats, g	19.1	15.2	16.8	5.3, 32.6	22.4	9.0	20.3	13.6, 35.2	0.02	0.59
Monounsaturated Fats, g	21.9	21.0	15.7	6.1, 38.8	21.7	8.0	20.2	13.7, 33.6	0.34	0.46
Polyunsaturated Fats, g	12.3	16.8	8.5	2.9, 20.5	15.4	7.1	13.9	8.4, 28.6	<0.001	0.42
Calcium, mg	854.6	549.5	659.1	292.3, 1654.7	930.2	367.0	832.9	574.4, 1499.1	0.14	0.32
Vitamin C, mg	120.3	93.8	91.7	28.5, 237.0	75.9	46.0	68.5	30.6, 125.6	<0.01	0.16
Fiber, g	17.8	11.2	14.9	5.2, 32.2	18.3	7.6	17.0	9.7, 27.7	0.62	0.36
Iron, mg	11.5	9.1	9.3	3.2, 21.0	15.2	6.8	14.2	9.2, 22.2	<0.001	0.69
Whole Grains, oz	1.3	1.4	1.0	0.14, 2.8	0.91	1.0	0.5	0, 2.4	0.11	0.11
Vegetables, cups	1.7	1.3	1.5	0.49, 2.9	1.4	0.82	1.3	0.63, 2.5	0.10	0.56
Fruits, cups	1.1	0.90	0.7	0.20 2.3	0.98	0.72	0.89	0.13, 1.9	0.72	0.32
Dairy, cups	1.8	1.5	1.2	0.36, 3.9	1.7	0.94	1.5	0.61, 3.0	0.83	0.46
Sugar, g	76.9	46.6	69.7	27.4, 143.4	87.2	36.3	82.7	49.2, 129.0	0.09	0.50

kcal: kilocalories; g: grams; mg: milligrams; oz: ounce equivalents; * *p*-value testing difference between means from FFQ and 3-day dietary records.
